# A novel mutation in the ABCC8 gene causing maturity-onset diabetes of the young: A case report

**DOI:** 10.1016/j.clinme.2024.100033

**Published:** 2024-03-20

**Authors:** Yifan Guo, Yangli Chen, Wen Liang, Lirong Zeng, Fang Hu, Yingjuan Zeng, Li Cong

**Affiliations:** Department of Endocrinology and Metabolism, The Fifth Affiliated Hospital Sun Yat-sen University, Zhuhai, Guangdong, China

**Keywords:** Maturity-onset diabetes of the young (MODY), ABCC8, Variant, Glucose, Case report

## Abstract

A 34-year-old woman was diagnosed with type 1 diabetes mellitus and treated with insulin for 24 years. The patient has a family history of diabetes in three consecutive generations. Her Whole exon sequencing showed a heterozygous mutation in the ABCC8 gene, and it also found some of her relatives to carry this mutation. She was diagnosed with MODY12 and received glimepiride therapy with the achievement of good glycaemic control.

## 1 Introduction

Maturity-onset diabetes of the young (MODY) is a mild familial diabetes mellitus with dominant inheritance, characterized by pancreatic β-cell destruction and disrupted insulin secretion.^1^ The pancreatic β-cell-expressed sulfonylurea receptor 1 (SUR1) and Kir6.2, encoded by ATP Binding Cassette Subfamily C Member 8 (ABCC8) and Potassium Inwardly Rectifying Channel Subfamily J Member 11 (KCNJ11) respectively, compose the pancreatic β-cell ATP-sensitive potassium (KATP) channel that triggers insulin release.^2^ The gain-of-function of ABCC8 is related to neonatal diabetes mellitus (ND) and MODY 12,^3^ most of which can be treated with sulfonylurea.^4^ Although MODY accounts for 1–4% of paediatric diabetes,^5^ misdiagnosis still results in many young people being treated unnecessarily with insulin. Here, we reported a novel ABCC8 mutation in a family of MODY 12.

### 1.1 Case presentation

The proband is a 34-year-old woman who was diagnosed with diabetes 24 years earlier. She has a family history of diabetes, and her mother, maternal grandmother, and maternal uncle had also been diagnosed with diabetes. When she was 10 years old, diabetic symptoms such as polyuria, polydipsia and weight loss have occurred and the fasting blood glucose (FBG) concentration >10.0 mmol/L (normal 3.60–6.10). She has been diagnosed with type 1 diabetes mellitus (T1DM) and has received insulin treatment for years. At 31 years old, the patient's body mass index was 18.2 kg/m^2^ (height, 153.0 cm; weight, 42.5 kg). The laboratory test results at the time of hospitalization showed that FBG concentration was 6.10 mmol/L; the HbA1c level was 6.10% (normal 4.0%–6.0%). Her kidney and liver function were both normal, with a glomerular filtration rate of 187.53 mL/min/1.73 m^2^ and liver function tests within the normal range. Her fasting C-peptide level was 359.20 pmol/L (normal 370.00–1,470.00), which was low but still could be detected. In the 75 g oral glucose tolerance test (OGTT), C-peptide level at 3 h after glucose gavage rose continuously (1,710.00 pmol/L). The T1DM specific autoantibodies, including the glutamic acid decarboxylase antibody (GADA), insulin autoantibody (IAA), and anti-islet cell antibody (ICA) were negative.

### 1.2 Mutation analysis

**Fig. 1:**
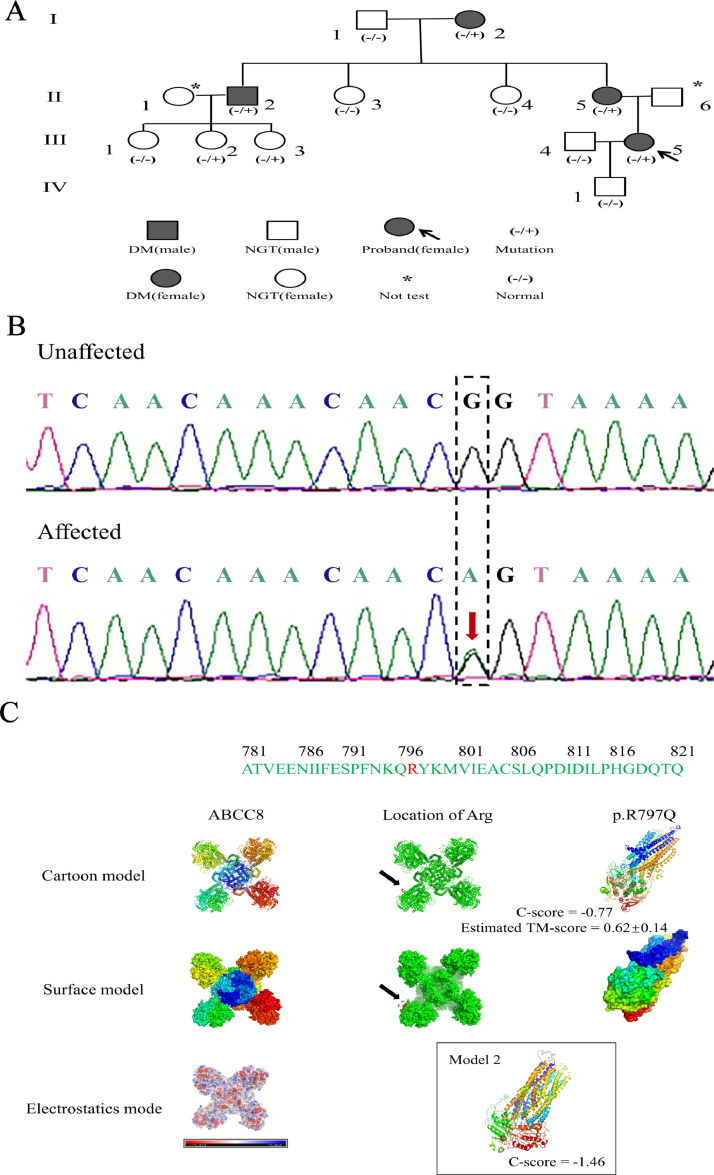
Pedigree of the family. Sanger sequencing of DNA. Mutation validation results showed that the sequence on the coding chain: G was replaced by A. 3D-structure model of the variant (The ABCC8 protein structure is shown in distinct colour. The location of the mutation p.R797Q is marked in red in the green colour scheme. Partial protein structure disruption caused by the missense mutation p.R797Q is shown in distinct colour. The models are predicted by I-TASSER (include model 1 and model 2). C-score is typically between [−5, 2], where a C-score of a higher value signifies a model with a higher confidence. Estimated TM-score > 0.5 shows a model of correct topology (model 1 C-score = −0.77; model 2 C-score = −1.46; model 1 TM-score = 0.62 ± 0.14)).

Abbreviation: DM, diabetes mellitus; NGT, normal glucose tolerance.

Whole exon sequencing (WES) of the proband found a heterozygous mutation in the ABCC8 gene: c.2390G> A, p. Arg797Gln, and such variants are inherited in an autosomal dominant manner. The proband's mother (II-5), maternal grandma (I-2), maternal uncle (II-2), as well as two cousins (III-2, 3) were found to carry the mutation in the ABCC8 (Fig. 1a). We also verified this mutation in ABCC8 by Sanger sequencing of all carriers (Fig. 1b). The mutations were a new mutation, which was undetected in the 1000Genome Project database and the GnomAD exon database. Referring to the American College of Medical Genetics and Genomics/Association for Molecular Pathology guidelines, the original amino acid (p. Arg797Gln) of the mutated region was determined to be pathogenic. Therefore, we consider this mutation of ABCC8 is highly probable to be a pathogenic mutation.

Protein Structures. After modelling this variant and predicting the structure and function of the mutated protein, the models suggested that the missense mutation resulted in a profound disruption of the protein structure (Fig. 1c).

### 1.3 Clinical treatment and follow-up

The proband was received acarbose therapy with a dose of 50 mg three times daily at 10 years old. Then her drug has been changed soon to biphasic premixed insulin aspartate 30 with a dose of 15 IU twice daily for years. Until 2,019, after diagnostic WES she was diagnosed with MODY12 in our hospital and received glimepiride and basal insulin glargine therapy. The patients were followed through May 2022 and experienced very good glycaemic control (HbA1c 6.0%).

## 2 Discussion

In this study, we reported on a MODY family with a new missense variant in exome 19 of the ABCC8 gene (c.2390G> A, p. Arg797Gln), which was detected for the first time in human. The proband was diagnosed with diabetes at age 10 years, while most evidence did not distinguish between the major types of diabetes. Considering normal BMI, childhood-onset diabetes and ineffective oral acarbose treatment, diagnosis of T2DM were ruled out. Although her T1DM specific antibodies were negative, a further 3.2% of T1DM were probably antibody negative T1DM within 3 years of diagnosis.^6^ Only her grandmother had been diagnosed with diabetes at that time, which may be directly causal for misdiagnosis. Her mother was misdiagnosed with T2DM at 38 years old and the age of onset was significantly later than that for the proband. With the presence of diabetes in three generations of this family, the proband underwent diagnostic WES and finally diagnosed with MODY12.

Most MDOY patients were treated with sulfonylureas but not insulin,^7^ because sulfonylureas could close the KATP channel and cause insulin release. However, sulfonylureas therapy alone has a poor therapeutic effect in our case, which differed from most previous reports. Long disease course is a probable reason for this discrepancy, and serum C–peptide levels mirror the loss of islet function. This mutation affects the ABC transporter domain but not the ABC transmembrane domain.^8^ We speculated this might associate with a faster rate of deterioration of β–cell function and poorer response to sulfonylureas. This supports that genetic testing is vital important for suspected cases of MODY, and sulfonylureas therapy should be started as early as possible after diagnosis.

In conclusion, our study was the first to report the novel missense variant of the ABCC8 gene (c.2390G> A) in a Chinese MODY 12 family. This report expanded the locus information of the ABCC8 mutations and could be helpful in an accurate diagnosis of MODY12.

## Funding

No funding was available for this study.

## Patient consent

We certify all methods were performed under relevant guidelines and regulations. All protocols were approved by the Medical Ethics Committee of The Fifth Affiliated Hospital Sun Yat-sen University. Informed consent was obtained from the patient.

## CRediT authorship contribution statement

**Yifan Guo:** Conceptualization, Formal analysis, Investigation, Project administration, Software, Visualization, Writing – original draft, Writing – review & editing. **Yangli Chen:** Data curation, Formal analysis, Project administration, Visualization. **Wen Liang:** Data curation, Resources, Visualization. **Lirong Zeng:** Data curation. **Fang Hu:** Investigation, Resources, Software. **Yingjuan Zeng:** Investigation, Supervision. **Li Cong:** Conceptualization, Supervision, Validation.

## Declaration of competing interests

The authors declare that they have no competing interests.

## Appendix Supplementary materials

Image, application 1
