# Supplementary material for: A novel mutation in the ABCC8 gene causing maturity-onset diabetes of the young: A case report
Source: Clin Med (Lond). 2024 Mar 20;24(2):100033. doi: 10.1016/j.clinme.2024.100033 (PMC11091440; doi:10.1016/j.clinme.2024.100033)
Supplement: Supplementary file 1 [file mmc1.docx]

Methods

Blood samples were collected from the subjects after obtaining informed consent. DNA library was prepared with MGIEasy regents (BGI, Shenzhen, China), then enriched for the coding exons of targeted genes using BGI V4 chip (BGI, Shenzhen, China), such sequencing libraries were performed on MGISEQ-2000 platform (BGI, Shenzhen, China).

Fastq files were assessed for quality control using the FastQC program, and the median sequencing depth was ≥100X for each sample, and coverage breadth was >95% at 1X coverage or higher. The BWA software was used to compare the hg19 version of the human genome reference sequence provided by UCSC, and the SNV and InDel mutations were found through the HaplotypeCaller of GATK. Mutation was detected in the 1000Genome Project database (http://www.internationalgenome.org/) and the GnomAD exon database (http://gnomad.broadinstitute.org). The pathogenicity was evaluated using the general American College of Medical Genetics and Genomics/Association for Molecular Pathology (ACMG/AMP) guidelines(11).

Sanger sequencing was performed on the sample from the proband’s relatives to confirm the pathogenic variant in the proband. Two primers (forward primer: TGTAGTAAGAGCGCAGTCGATG; reverse primer: CTGAGAACAAGCCCCTGAGAAT) were used to verify the region of ABCC8 gene mutation.

ABCC8 protein coding mutations were modeled using iTasser (https://zhanggroup.org/ITASSER/) and structure figures were prepared with PyMOL (The PyMOL Molecular Graphics System, Schrödinger, LLC).
